# Repeated influenza vaccination induces similar immune protection as first‐time vaccination but with differing immune responses

**DOI:** 10.1111/irv.13060

**Published:** 2022-10-21

**Authors:** Beiwei Ye, Liumei Shu, Yuanyuan Pang, Yaxin Guo, Yuanyuan Guo, Kexin Zong, Cong Chen, Xianzhi Zheng, Jie Zhang, Maoshun Liu, Xiaoju Yuan, Yingze Zhao, Danni Zhang, Dayan Wang, Changjun Bao, Jun Zhang, Liling Chen, George F. Gao, William J. Liu

**Affiliations:** ^1^ NHC Key Laboratory of Biosafety, National Institute for Viral Disease Control and Prevention Chinese Center for Disease Control and Prevention (China CDC) Beijing China; ^2^ Department of Health Care Beijing Daxing District Hospital Beijing China; ^3^ Suzhou Municipal Center for Disease Control and Prevention Suzhou Jiangsu China; ^4^ Department of Epidemiology, School of Public Health, Cheeloo College of Medicine Shandong University Jinan Shandong China; ^5^ Changzhou Center for Disease Control and Prevention Changzhou Jiangsu China; ^6^ School of Laboratory Medicine and Life Sciences Wenzhou Medical University Wenzhou Zhejiang China; ^7^ Jiangsu Provincial Center for Disease Control and Prevention Nanjing Jiangsu China; ^8^ CAS Key Laboratory of Pathogen Microbiology and Immunology, Institute of Microbiology Chinese Academy of Sciences (CAS) Beijing China; ^9^ Research Unit of Adaptive Evolution and Control of Emerging Viruses Chinese Academy of Medical Sciences Beijing China

**Keywords:** HAI titers, immune protection, influenza, repeated vaccination, vaccines

## Abstract

**Background:**

Recent seasonal epidemics of influenza have been caused by human influenza A viruses of the H1N1 and H3N2 subtypes and influenza B viruses. Annual vaccination is recommended to prevent infection; however, how annual influenza vaccination influences vaccine effectiveness is largely unknown.

**Methods:**

To investigate the impact of repeated vaccination on immune and protective effect, we performed a prospective seroepidemiologic study. Participants with or without prior vaccination (2018–2019) were enrolled during the 2019–2020 influenza season. Inactivated quadrivalent influenza vaccine (IIV4) was administered through the intramuscular route, and venous blood samples were collected regularly to test hemagglutination inhibition (HAI) titers.

**Results:**

The geometric mean titers and proportion with titers ≥40 against the influenza vaccine components peaked at 30 days post‐vaccination. At Day 30, the geometric mean titer and proportion with titers ≥40 in participants who had been previously vaccinated were higher for H3N2 but similar for both B lineages (Victoria and Yamagata) as compared with participants vaccinated for the first time. As for H1N1, the geometric mean titer was lower in repeated vaccinated participants, but the proportion with titers ≥40 was consistent in both groups.

**Conclusions:**

Repeated vaccination provides similar or enhanced protection as compared with single vaccination in first‐time vaccinees.

## 1 INTRODUCTION

Influenza is an acute and highly contagious viral infection characterized by acute respiratory symptoms and can induce substantial morbidity and mortality in humans.^1^ Seasonal epidemics of influenza, mainly caused by influenza A viruses (IAVs) of the H1N1 and H3N2 subtypes and influenza B viruses (IBVs) of Victoria and Yamagata lineages, typically kill 290,000–650,000 people each year worldwide,^2^, ^3^ thus posing a significant threat to global public health.

Vaccination remains the most effective method for prevention and control of infection.^4^ Due to the characteristic changes and uncertainty in the hemagglutinin (HA) and neuraminidase (NA) glycoproteins,^3^, ^5^ currently‐used seasonal influenza vaccines are regularly updated. The Global Influenza Surveillance and Response System, established by the World Health Organization (WHO), makes recommendations on vaccine strains for the Northern and Southern hemispheres 6–8 months before the influenza season starts to combat antigenic drift in the circulating influenza subtypes.^6^

Since 2010, the Advisory Committee on Immunization Practices has recommended annual influenza vaccination for all persons aged ≥6 months who do not have contraindications.^7^ However, this recommendation has faced the question of how prior vaccination may influence the immune response to the current vaccine. Previous researches have suggested that repeated annual influenza vaccination can blunt future vaccine‐induced antibody responses, particularly to IAV H3N2 strains,^8^, ^9^, ^10^, ^11^ raising the concern that frequent vaccination may impair vaccine immunogenicity and effectiveness. On the contrary, other studies of influenza vaccines indicated that residual protection against influenza viruses occurred even though the predominant viruses were antigenically distinct from the previous season's vaccine components.^12^, ^13^

In this study, we compared antibodies characterized by hemagglutination inhibition (HAI) titer in individuals vaccinated for two consecutive years with those who received vaccine in the current season only, evaluating whether vaccination history influences antibody response induced by inactivated, quadrivalent split‐virion influenza vaccine (IIV4). This study was a prospective observational cohort, designed to examine the similarities and differences in the humoral immune response between individuals with different influenza vaccination histories.

## 2 MATERIALS AND METHODS

### 2.1 Participant enrollment

Subjects were enrolled using random cluster sampling.^14^ Exclusion criteria included documented contraindications to receipt of inactivated influenza vaccine, immunosuppressive treatment or immunocompromised condition, Guillain–Barré syndrome, dementia, or Alzheimer's disease. Eligible participants aged 4–81 years who had not yet received the 2019–2020 influenza vaccine were enrolled from November to early December 2019. All participants received the 2019–2020 standard‐dose IIV4 containing A/Brisbane/02/2018 (H1N1)pdm09, A/Kansas/14/2017(H3N2), B/Colorado/06/2017 (Victoria lineage), and B/Phuket/3073/2013 (Yamagata lineage). Written informed consent was obtained from all adult participants and guardians of minors at enrollment. Upon enrollment, every participant completed a questionnaire that included demographic information, employment category, medical history, and influenza vaccination history. This study was approved by the Ethics Review Committee of National Institute for Viral Disease Control and Prevention, Chinese Center for Disease Control and Prevention (IVDC2020‐022).

### 2.2 Sample collection

Pre‐vaccination serum samples were collected on December 9, 2019, and then the IIV4 injections were administered on the same day. Post‐vaccination sera were collected at 7, 30, and 245 days after vaccination. Samples were aliquoted and frozen at −80°C until assayed. Participants were instructed to report influenza‐like illness (ILI). Participants who had an axillary temperature of 37.8°C or higher and at least one respiratory (cough, pharyngitis, or dyspnea) or systemic (chills, headache, malaise, or myalgia) symptom were asked to self‐report. If ILI was reported, a throat swab was taken within 24 h after onset of symptoms, which was used for influenza virus detection and subsequent subtyping of IAV and IBV by real‐time Reverse Transcription Polymerase Chain Reacation (RT‐PCR).

### 2.3 Antigenic components

All participants received a single 0.5 ml intramuscular injection of the quadrivalent vaccine for 2019–2020 season in the deltoid muscle. These vaccines contain 15 μg of HA antigen from the following virus strains: an A/Brisbane/02/2018(H1N1) pdm09‐like virus, an A/Kansas/14/2017(H3N2)‐like virus, a B/Colorado/06/2017‐like virus (B/Victoria/2/87 lineage), and a B/Phuket/3073/2013‐like virus (B/Yamagata/16/88 lineage). According to WHO recommendations on influenza vaccine composition, quadrivalent vaccines for use in the 2018–2019 Northern hemisphere influenza season contain two different components, for IAVs, an A/Michigan/45/2015(H1N1)pdm09‐like virus and an A/Singapore/INFIMH‐16‐0019/2016(H3N2)‐like virus.

### 2.4 HAI test

Four agglutination unit antigens were prepared from chicken embryo‐adapted vaccine strains, A/Brisbane/02/2018(H1N1)pdm09, A/Kansas/14/2017(H3N2), B/Colorado/06/2017 (Victoria lineage), and B/Phuket/3073/2013 (Yamagata lineage). The virus strains used for HAI testing were stored in multiple aliquots at −80°C. HAI assays were performed with pre‐ and post‐vaccination serum specimens using 1% turkey erythrocytes. Sera treated with receptor destroying enzyme and adsorbed on guinea pig or chicken red blood cells before the test were diluted twofold starting from a 1:10 dilution. HAI titers were defined as the highest serum dilution that completely inhibited hemagglutination and were recorded as 1:5 when below 1:10.^15^

### 2.5 Outcomes measures

The outcomes were serologic responses to egg‐grown vaccine reference viruses by HAI test for A/H1N1, A/H3N2, B/Victoria, and B/Yamagata vaccine pre‐ and post‐vaccination using the following measures: geometric mean titers (GMTs), seroconversion rate (SCR), and the proportion with titers ≥1:40. Seroconversion is an increase in the antibody titer chosen to indicate an immune response. SCRs were defined as the proportion of participants with either a pre‐vaccination titer of <1:10 and 30 days post‐vaccination titer ≥1:40 or a pre‐vaccination titer ≥1:10 and at least fourfold rise between pre‐ and post‐vaccination titers.^16^, ^17^, ^18^ Besides, a post‐vaccination titers ≥ 1:40 is generally accepted to correspond to a 50% reduction in the risk of contracting influenza in a susceptible population.^19^, ^20^ The proportion of participants with titers ≥1:40 was used as another outcome measure.

### 2.6 Statistical analysis

Statistical analysis was performed using IBM spss version 24. Statistical significance was assumed when *p* < 0.05. Statistical tests included Student's *t* test for continuous variables, non‐parametric tests for discrete variables and skewed distribution data, and chi‐square test for categorical variables. Data analyzed in this paper are available from the corresponding author on reasonable request.

## 3 RESULTS

### 3.1 Participants and baseline characteristics

**FIGURE 1:**
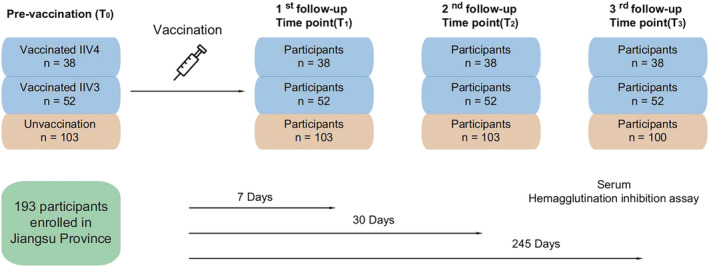
Monitoring flow chart. One hundred and ninety‐three participants were recruited for this study. Pre‐vaccination sera were collected on December 9, 2019, and the IIV4s injections were administered on the same day (T_0_). Post‐vaccination sera were collected at 7 (T_1_), 30 (T_2_), and 245 (T_3_) days after vaccination. Three participants in the single vaccination group dropped out before the fourth blood sample was collected. Abbreviation: IIV3, inactivated, trivalent split‐virion influenza vaccine; IIV4, inactivated, quadrivalent split‐ virion influenza vaccine

Enrollment began in November 2019 and continued through the following month, recruiting volunteers from several communities of Jiangsu who had not yet received the influenza vaccine in the 2019–2020 season. A total of 193 subjects were eligible, including 38 participants who were vaccinated with inactivated, quadrivalent split‐virion influenza vaccines (IIV4s) in 2018–2019; 52 participants vaccinated with inactivated, trivalent split‐virion influenza vaccines (IIV3s) in 2018–2019; and 103 participants who were unvaccinated in 2018–2019. Individuals were divided into two groups according to influenza vaccination status. The prior vaccination group included participants vaccinated with IIV3s/IIV4s in 2018–2019 followed by receipt of IIV4s in 2019–2020 under the study protocol, whereas the single vaccination group included participants unvaccinated in 2018–2019 followed by vaccination with IIV4s in 2019–2020 during this study. All participants provided a pre‐vaccination blood specimen and received vaccination on the same day. The post‐vaccination sera were collected at 7 days, 30 days, and 245 days after vaccination. At the time of final blood sampling, all participants in the prior vaccination group completed this study, whereas three participants were lost to follow‐up in the single vaccination group (Figure 1). During the study, 25 individuals reported ILI symptoms, and two had laboratory‐confirmed symptomatic influenza, infected with H3N2 and B/Colorado Victoria lineage, respectively.

There were 78 males and 115 females enrolled in this study, ranging from 4 to 81 years of age. The median age of participants was 41 years in the prior vaccination group and 44 years in the single vaccination group. There were no significant differences in distribution between the two groups in terms of sex (*p* = 0.686) or age (*p* = 0.894). Chronic diseases and exposure to cigarette smoke were expected to be risk factors for severe disease or complications induced by influenza.^21^, ^22^, ^23^ The proportion of individuals who suffered from chronic diseases in the prior vaccination group was 19.1% (18/90), which was not significantly different from 16.5% (17/103) in the single vaccination group (*p* = 0.530). Sixteen participants (17.8%) had a history of smoking in the prior vaccination group, including current and former smokers, which was approximately the same proportion as in the single vaccination group (16.5%, 17/103, *p* = 0.815).

### 3.2 GMTs of antibodies after vaccination

**FIGURE 2:**
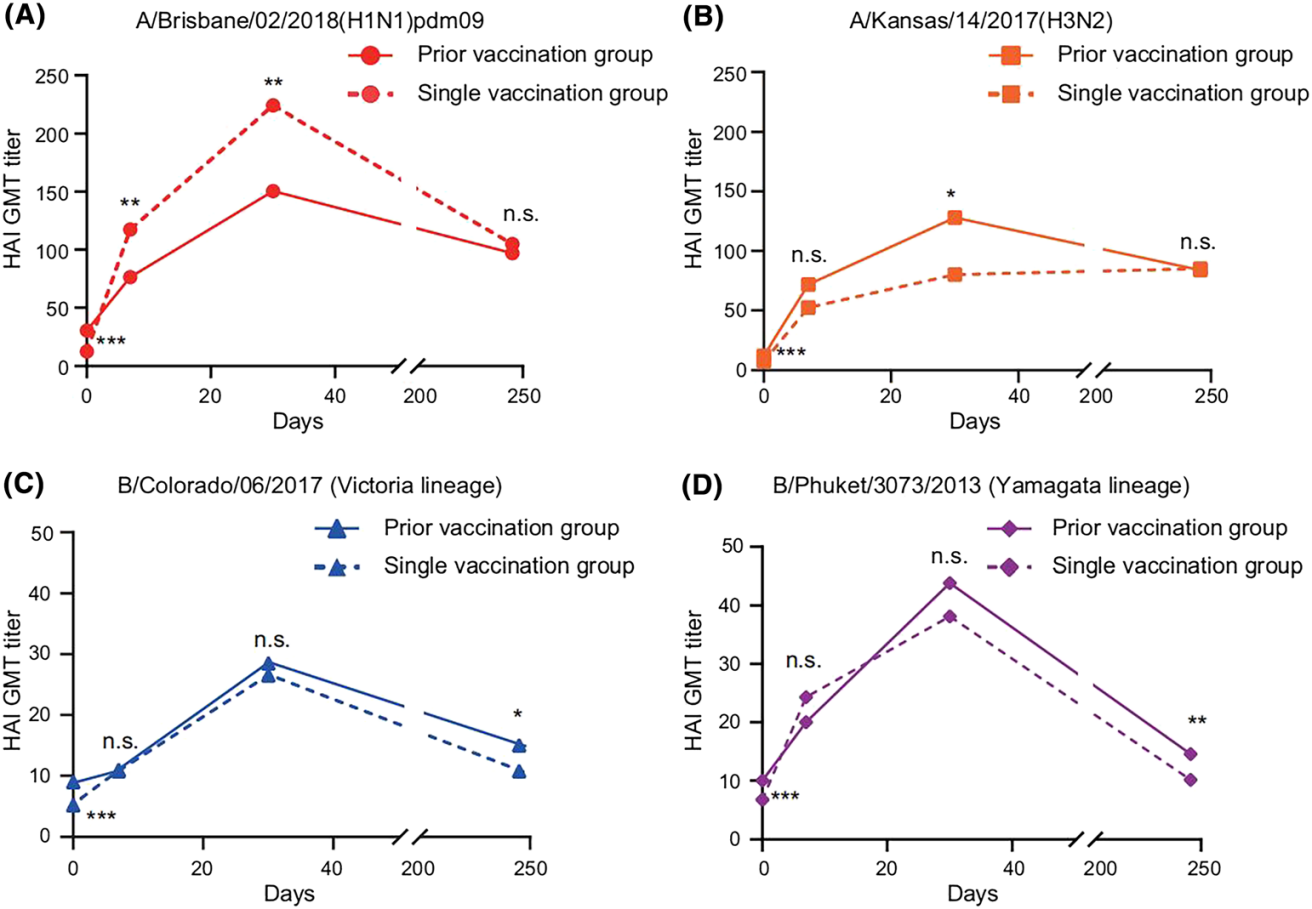
Trends in hemagglutination inhibition (HAI) titers against each influenza vaccine component. HAI assays were performed before and 7, 30, and 245 days after inactivated, quadrivalent split‐virion influenza vaccines (IIV4s) vaccination in the 2019–2020 season. HAI responses against the hemagglutinin (HA) of the indicated vaccine viruses, A/Brisbane/02/2018 (H1N1)pdm09, A/Kansas/14/2017 (H3N2), B/Colorado/06/2017 (Victoria lineage), and B/Phuket/3073/2013 (Yamagata lineage), are shown. The solid line indicates the prior vaccination group, including participants vaccinated in both 2018–2019 and 2019–2020. The dashed line indicates the single vaccination group, including participants vaccinated in 2019–2020 only. n.s. indicates *p* ≥ 0.05; *p < 0.05; **p < 0.01; ***p < 0.001. Abbreviation: GMT, geometric mean titer

The immune response profile following vaccination was analyzed by HAI test in pre‐ and post‐vaccination sera of the volunteers. Dynamic changes in immunogenicity could be observed among the vaccinees before and after vaccination during the study period in 2019–2020 (Figure 2 and Table 1). Before vaccination, the GMT in the prior vaccination group was higher than that in the single vaccination group for each influenza vaccine component. Although IIV3 in 2018–2019 did not cover B/Yamagata lineage, the GMT against B/Yamagata was still statistically higher in the prior vaccination group before vaccination in 2019–2020 (*p* = 0.017). Serum HAI antibodies to each of the four vaccine strains in both groups increased rapidly after vaccination and then decreased again toward the end of the influenza season. For every vaccine strain, Mauchly's test of sphericity indicated that there was a remarkable difference in the HAI titers among four sampling times. It also indicated that the GMT trends from pre‐vaccination to post‐vaccination were significantly different between the prior vaccination group and the single vaccination group. However, serological responses varied by vaccine strain after vaccination, with greater change in post‐vaccination GMTs against influenza A vaccine strains, A/Brisbane (H1N1) and A/Kansas (H3N2), and more modest variations for B/Colorado (Victoria lineage) and B/Phuket (Yamagata lineage).

**TABLE 1:** The geometric mean titers pre‐ and post‐vaccination for individuals in Jiangsu province, 2019–2020

Time	A/H1N1		*p* value	A/H3N2		*p* value	B/Victoria		*p* value	B/Yamagata^a^		*p* value
	Prior vaccination group (*n* = 90)	Single vaccination group (*n* = 103)		Prior vaccination group (*n* = 90)	Single vaccination group (*n* = 103)		Prior vaccination group (*n* = 90)	Single vaccination group (*n* = 103)		Prior vaccination group (*n* = 90)	Single vaccination group (*n* = 103)	
0	30.314	12.656	<0.001***	28.167	11.845	<0.001***	14.889	5.437	<0.001***	19.056	8.398	<0.001***
7	76.387	117.403	0.009**	180.000	155.971	0.119	17.000	15.728	0.821	39.056	45.583	0.189
30	150.439	224.002	0.004**	247.222	186.117	0.042*	55.278	53.398	0.776	68.000	65.291	0.438
245	96.986	104.831	0.448	144.944	162.150	0.998	29.333	20.800	0.017*	22.000	16.050	0.001**

a Trivalent inactivated influenza vaccine (IIV3) used in the 2018–2019 influenza seasons did not contain Yamagata lineage.

* p < 0.05.

** p < 0.01.

*** p < 0.001.

After vaccination, the GMT for A/H1N1 in the prior vaccination group was consistently lower than that in the single vaccination group (Figure 2). The differences were statistically significant at Day 7 (*p* = 0.009) and Day 30 (*p* = 0.004) but not at Day 245 (*p* = 0.448). For A/H3N2, the GMT in the prior vaccination group was slightly higher than that in the single vaccination group with a statistical difference at Day 30 (*p* = 0.042). For B/Victoria and B/Yamagata, the GMT differences between the two groups were not statistically significant at Day 7 (*p* = 0.821; *p* = 0.189) or Day 30 (*p* = 0.776; *p* = 0.438) but reached statistical significance at Day 245 (*p* = 0.017; *p* = 0.001). There was a significant decrease in GMT for each subtype and lineage at 245 days post‐vaccination, but HAI antibody titers against influenza A remained above 40.

### 3.3 Immune protection

To further evaluate the efficacy of seasonal influenza vaccines, we compared SCRs and the proportion with titers ≥40 between the two vaccination groups. SCRs against A/H1N1 were lower in the prior vaccination group than in the single vaccination group, and the differences were statistically significant (48.89% vs 84.47%, *p* < 0.001). SCRs post‐vaccination against A/H3N2, B/Victoria, and B/Yamagata were largely consistent between the two groups (Table 2).

**TABLE 2:** Comparison of seroconversion rates of seasonal influenza antibodies in Jiangsu province between different groups, 2019–2020

Item	Prior vaccination group (vaccinated IIVs in 2018 and 2019)		Single vaccination group (vaccinated IIVs in 2019)		χ^2^	*p*
A/H1N1	48.89%	(44/90)	84.47%	(87/103)	27.881	<0.001**
A/H3N2	82.22%	(74/90)	73.79%	(76/103)	1.974	0.160
B/Victoria	43.33%	(39/90)	49.51%	(51/103)	0.737	0.390
B/Yamagata	53.33%	(48/90)	56.31%	(58/103)	0.172	0.678

Abbreviation: IIVs, inactivated split‐virion influenza vaccines.

* p < 0.05.

** p < 0.01.

*** p < 0.001.

Given that the seroconversion influenced by the baseline GMT and the GMTs among participants in the prior vaccination group was significantly higher than the other group, we compare the percentage of vaccinees achieving HAI titers ≥40 in both groups, which is generally accepted to correspond to a 50% reduction in the risk of contracting influenza in a susceptible population.^19^, ^24^

Baseline proportion with titer ≥40 in the prior vaccination group was higher than that in the single vaccination group with significant differences for A/H1N1 (54.44% vs 26.21%, *p* < 0.001), A/H3N2 (17.78% vs 7.77%, *p* = 0.036), and B/Victoria (11.11% vs 0.00%, *p* = 0.002). Of note, baseline proportions with titer ≥40 for A/H3N2, B/Victoria, and B/Yamagata in both groups were consistently lower than that for A/H1N1. For all four vaccine viruses, the proportion with titer ≥40 increased rapidly after vaccination and peaked on Day 30, but the rates of increase were different (Table 3). The percentage of participants with an HAI titer ≥40 for the IAVs increased more rapidly compared with IBVs. At the peak, the percentage increased to more than 90% among all participants for A/H1N1, about 80% for A/H3N2, 50% for B/Victoria, and more than 60% for B/Yamagata. Thereafter, the proportion decreased as GMT declined. For A/H1N1 and A/H3N2, the percentage of participants with an HAI titer ≥40 remained higher than 80% at Day 245, although the GMT was significantly lower than at the peak. However, there were significant decreases in the proportions with titer ≥40 for B/Victoria and B/Yamagata from Day 30 to Day 245, and the proportions for B/Victoria and B/Yamagata on Day 245 were less than 30%. Finally, there were roughly the same proportions with titer ≥40 against the four vaccine viruses between the two groups post‐season, except for the higher percentage with titer ≥40 for A/H3N2 in the prior vaccination group on Day 30.

**TABLE 3:** Comparison of the proportion with titers ≥40 of seasonal influenza antibodies in Jiangsu province between different groups, 2019–2020

Item	Prior vaccination group (vaccinated IIVs in 2018 and 2019)		Single vaccination group (vaccinated IIVs in 2019)		χ^2^	*p*
Day 0						
A/H1N1	54.44%	(49/90)	26.21%	(28/103)	16.036	<0.001***
A/H3N2	17.78%	(16/90)	7.77%	(8/103)	4.421	0.036*
B/Victoria	11.11%	(10/90)	0.00%	(0/103)	9.914	0.002**
B/Yamagata	6.67%	(6/90)	3.88%	(4/103)	0.297	0.586
Day 7						
A/H1N1	84.44%	(76/90)	84.47%	(87/103)	0.000	0.997
A/H3N2	74.44%	(67/90)	64.08%	(66/103)	2.409	0.121
B/Victoria	12.22%	(11/90)	16.50%	(17/103)	0.710	0.399
B/Yamagata	34.44%	(31/90)	45.63%	(47/103)	2.496	0.114
Day 30						
A/H1N1	93.33%	(84/90)	92.23%	(95/103)	0.086	0.769
A/H3N2	90.00%	(81/90)	77.67%	(80/103)	5.280	0.022*
B/Victoria	50.00%	(45/90)	49.51%	(51/103)	0.005	0.946
B/Yamagata	70.00%	(63/90)	61.17%	(63/103)	1.654	0.198
Day 245						
A/H1N1	90.00%	(81/90)	88.00%	(88/100)	0.151	0.698
A/H3N2	81.11%	(73/90)	80.00%	(80/100)	0.037	0.847
B/Victoria	26.67%	(24/90)	22.00%	(22/100)	0.562	0.453
B/Yamagata	18.89%	(17/90)	13.00%	(13/100)	1.235	0.266

* p < 0.05.

** p < 0.01.

*** p < 0.001.

## 4 DISCUSSIONS

In China, the estimated general influenza vaccination coverage for 1.4 billion Chinese people in 2019 is merely around 2%, far behind that for people from American and some other countries and regions.^25^ People's unwillingness to receive influenza vaccine can be partly attributed to concern on the benefits of annually influenza vaccination. While sophisticated statistical models have been used to account for the outcomes of repeated influenza vaccination,^12^, ^13^ only a small fraction of the published studies tracked antibody changes throughout the whole season from the same subjects. Our study was a prospective observational cohort based on the Chinese population, including people of all ages, recording changes in HAI titers from vaccination to approximately 8 months after vaccination.

This study reported the serological response to influenza vaccination among people with different histories of prior vaccination. By analyzing HAI antibodies titers against influenza vaccine strains, we observed substantial variability in baseline GMTs, proportions with titers ≥40, and the magnitude of vaccination responses. Serum antibody levels increased rapidly after vaccination, and there was a significant reduction 245 days post‐vaccination, especially for IBV components. The majority of studies have also demonstrated that HAI titers wane with time following influenza vaccination or infection and are unlikely to persist year‐round.^14^, ^26^, ^27^, ^28^ Consistent with our findings, these conclusions support the annual vaccination strategy for current influenza vaccines.

Our study focused on the differences in protective effect between prior vaccination and single vaccination groups. The GMTs and proportions with titers ≥40 for B/Victoria and B/Yamagata were similar between the two groups, suggesting that vaccination continuity did not elicit any improvement or deterioration in immune protection against IBV. However, IIV4s elicited stronger immune responses to A/H3N2 in the prior vaccination group. Higher GMT and proportion with titers ≥40 for A/H3N2 at Day 30 in the prior vaccination group suggested that vaccination for two consecutive years may promote the protective effectiveness of the current‐season vaccine. The improved immune protection against A/H3N2 in the prior vaccination group may be conferred by residual protection and cross‐reactivity elicited by previous vaccines. Several studies have provided evidence of residual protection against A/H3N2 viruses from previous‐season vaccination.^12^, ^29^

However, consistent with other observations,^30^, ^31^, ^32^, ^33^ our results showed that prior seasonal vaccination appeared to negatively influence the strength of the immune response to A/H1N1 elicited by influenza vaccines. Whereas, the blunting GMT for A/H1N1 is not so concerning given the similar proportions with titer ≥40 between the two groups. The reduced GMT for A/H1N1 did not result in poor immune protection rates, which could provide adequate serological protection in the prior vaccination group.

There are many reasons for the discrepancy in immune response to A/H1N1 and A/H3N2. The influence of pre‐existing immunity on vaccine responses is complex. In addition to vaccination‐induced pre‐existing immunity, previous natural exposure to influenza viruses of a related subtype may afford some level of cross‐reactive immunity and retention of immune memory.^34^, ^35^, ^36^ Furthermore, antigenic components for IAVs in 2018–2019 were different from that in 2019–2020. The antigenic distance hypothesis is one potential explanation, which suggests that repeat vaccine efficacy can be influenced by antigenic distances among the influenza vaccines of previous and current seasons, and the current epidemic strain.^37^, ^38^ Sequence differences in influenza vaccine strains between two consecutive seasons could be one of the reasons for the discrepancy in these findings. Additional research on cross‐reactivity of antibodies and/or T‐cells between antigenically distinct viruses will be beneficial to understanding the mechanism behind whether pre‐existing immunity boosts or impairs reactive responses to the current influenza vaccine.

Actually, the concept of seroprotection can be defined as titer ≥40 within the vaccines as used in previous studies,^39^, ^40^, ^41^ although it is also indicated that titer ≥40 can only contribute 50% protection.^19^, ^24^ Herein, in our study, 25 participants (12.95%) had reported ILIs, and only two participants (1.03%) were laboratory‐confirmed as influenza cases. This infection proportion of vaccinees is lower than the previous studies.^42^ However, the ILIs surveillance in our study relied on self‐reported method. If participants had delayed or failed to report ILI symptoms, the incidence of influenza would have been underestimated. On the other hand, laboratory testing was limited to influenza viruses. Other respiratory viruses, such as adenovirus, rhinoviruses, human coronaviruses, and parainfluenza viruses, could be possible etiologies for ILIs.^43^, ^44^ Therefore, we evaluated the similarities and differences in immunogenicity mainly by HAI titers and proportions with titers ≥40 in our cohort.

These results are subject to several limitations. Our study included 193 individuals, and the sample size may be small in number. Nevertheless, our study was a prospective cohort study, and subjects were divided into the control group and the experimental group according to their vaccination histories. All participants received the 2019–2020 standard‐dose IIV4 at the same time and followed up at the same interval. The calculation indicates that at 5% type I error, the sample size of 190 (exclusions: three withdrew without contributing to the ILI‐reporting) was anticipated to provide 98.52% statistical power to detect a difference in proportion with titers ≥40^45^ between the two groups. Besides, the study only included vaccination data from two consecutive years, comparing immunity for several successive years may provide further insights into the analysis.

Overall, our study suggests that vaccination provides immune protection regardless of prior vaccination history. Consecutive influenza vaccinations can be effective in replenishing antibody levels and may be beneficial for the effectiveness of vaccination for the next season. This result provides evidence to support decision‐making for receiving the influenza vaccine annually. Based on the persistence of poor influenza vaccination rates in China,^25^ community health institutions should strengthen vaccine advocacy to increase immunization coverage. For vulnerable populations, community health care centers can establish health records and proactively remind individuals each year to receive their influenza vaccination ahead of the flu season.

## CONFLICT OF INTEREST

The authors declare no competing interests.

## AUTHOR CONTRIBUTIONS

**George F. Gao, William J. Liu, and Liling** **Chen** designed and supervised the study. **Beiwei Ye, Liumei Shu, Yuanyuan Pang, Yaxin Guo, Yuanyuan Guo, Kexin Zong, Cong Chen, Xianzhi Zheng, and Jie Zhang** collected the samples. **Beiwei Ye, Liumei Shu, Yaxin Guo, Yuanyuan Guo, Jie Zhang, Kexin Zong, and Maoshun Liu** conducted the experiments and analyzed data. **Beiwei Ye, Xiaoju Yuan, Yingze Zhao, and Danni Zhang** administrated the project. **Dayan Wang, Changjun Bao, and Jun Zhang** provided necessary resources for experiments. **Beiwei Ye, Liumei Shu, and William J. Liu** wrote the initial drafts of the manuscript. **Yuanyuan Pang and Liling Chen** commented on and revised drafts of the manuscript. All authors have approved the final version and agreed to be accountable for all aspects of the work.

### PEER REVIEW

The peer review history for this article is available at https://publons.com/publon/10.1111/irv.13060.

## Data Availability

The data that support the findings of this study are available on request from the corresponding author. The data are not publicly available due to privacy or ethical restrictions.
